# Overactive autophagy is a pathological mechanism underlying premature suture ossification in nonsyndromic craniosynostosis

**DOI:** 10.1038/s41598-018-24885-z

**Published:** 2018-04-25

**Authors:** Shanshan Qiu, Jing Wang, Siqi Huang, Shouqing Sun, Zhen Zhang, Nan Bao

**Affiliations:** 1 Department of Pediatric Surgery, Shanghai Children’s Medical Center, Shanghai Jiaotong University, School of Medicine, Shanghai, China; 2 Pediatric Translational Medicine Institute, Shanghai Pediatric Congenital Heart Disease Institute, Shanghai Children’s Medical Center, Shanghai Jiaotong University, School of Medicine, Shanghai, China

## Abstract

Nonsyndromic craniosynostosis (NSC) is the most common craniosynostosis with the primary defect being one or more fused sutures. In contrast to syndromic craniosynostosis, the etiopathogenesis of NSC is largely unknown. Here we show that autophagy, a major catabolic process required for the maintenance of bone homeostasis and bone growth, is a pathological change associated with NSC. Using calvarial suture mesenchymal cells (SMCs) isolated from the fused and unfused sutures of NSC patients, we demonstrate that during SMC differentiation, the level of the autophagosomal marker LC3-II increases as osteogenic differentiation progresses, particularly at differentiation day 7, a stage concurrent with mineralization. In fused SMCs, autophagic induction was more robust than that in unfused SMCs, which consequently led to enhanced mineralized nodule formation. Perturbation of autophagy with rapamycin or wortmannin promoted or inhibited the ossification of SMCs, respectively. Our findings suggest that autophagy is essential for the osteogenic differentiation of SMCs and that overactive autophagy is a molecular abnormality underlying premature calvarial ossification in NSC.

## Introduction

Craniosynostosis is a common congenital craniofacial malformation featuring the premature ossification of one or more cranial sutures, and with an incidence of 1 in 2000–2500 live births^1^. Nonsyndromic craniosynostosis (NSC) accounts for greater than 70% of craniosynostosis cases and the majority of these occur sporadically^2,3^. Although mutations in common syndromic craniosynostosis-associated genes and chromosomal aberrations have been found to be associated with NSC^4–6^, the molecular etiopathogenesis of NSC is largely unknown.

One salient feature of NSC is the normal development of the unaffected calvarial suture, albeit with the presence of premature ossification in the affected suture. The different phenotypes of calvarial sutures in the same genetic setting suggest that NSC defect is a locally-confined pathogenetic process. It has been proposed that local pathological changes are driven by the local alterations in the expression of genes involved in osteogenic signaling. Comparative analyses of gene expression in the fused and patent sutures of NSC patients identified differential gene expression between affected and unaffected sutures^7–10^. Elevated expression of LIM mineralization protein (LMP) and potent osteoinductive factors such as transforming growth factors and insulin-like growth factors have been reported to be associated with fused calvarial suture^11,12^. Mesenchymal stromal cells residing in fused sutures exhibit constitutively active osteogenic signaling and enhanced osteogenic potential^11^. The extracellular matrices produced from the fused and unfused sutures also exhibit differential ability to regulate the behavior of surrounding osteoblasts^13^. Although significant advances have been achieved in understanding the molecular pathogenesis of NSC, much has yet to be revealed.

There is a growing body of evidence suggesting that autophagy is a critical process for bone health and development. This process appears to be involved in the differentiation and homeostasis of both osteoblasts and osteoclasts^14–17^, and is thereby involved in various bone pathologies such as Paget’s disease^18^, osteoporosis^19^, osteoarthritis, and age-related bone loss^20,21^. The induction of autophagy has been shown to be a survival response to oxidative stress^22^, which helps to maintain the stemness of bone marrow-derived mesenchymal stromal cells^23^. In addition, it is required for the osteogenic differentiation of human mesenchymal stem cells/osteoblasts^14,24^. Other than its role in bone homeostasis, autophagy in chondrocytes also mediates the pro-growth effect of FGF signaling, an important regulatory pathway related to craniosynostosis^25^.

However, little is known regarding the role of autophagy in calvarial ossification and cranial bone diseases. Autophagy has been described in mandible-derived bone mesenchymal/stromal stem cells^26^, which are involved in intramembranous ossification similar to that observed in calvaria-derived bone mesenchymal stem cells. Interestingly, mandible-derived bone mesenchymal stem cells/multipotent stromal cells exhibit a higher level of autophagic activity and anti-aging capacities than those of tibia-derived bone mesenchymal stem cells^27^, which are involved in the endochondrial ossification process. This suggests that the intramembranous ossification process might be more dependent on autophagy. Therefore, it is likely that overactive autophagy is a pathological mechanism of premature suture ossification in NSC. In the current study, we provide the first evidence suggesting a direct role for autophagy in the calvarial ossification process, using calvarial SMCs isolated from the fused and unfused sutures of NSC patients. Results indicate that overactive autophagy is a likely mechanism underlying the pathogenesis of craniosynostosis.

## Materials and Methods

### Patients and specimens

All calvarial tissue fragments were collected from human donors undergoing cranial surgery for nonsyndromic monosutural craniosynostosis at the Shanghai Children’s Medical Center. The characteristics of patients participating in this study and the experiments for which samples were used are shown in Table 1. Patients were 7 to 14 months of age (mean 11.2 months). Only nonsyndromic monosutural patients were chosen for this study. A matched pair of suture tissues, consisting of one prematurely fused suture and one unfused suture, was taken from each patient. Written informed consent was obtained from all participants’ legal guardians. Ethical clearance was obtained from the Ethics and Research Committees of Shanghai Children’s Medical Center in China, and all experiments were performed in accordance with relevant guidelines and regulations.

**Table 1:** Patients and specimens.

Patient ID	Age (months)	Sex	Craniosynostosis type	Fused suture	Unfused suture	Experiments
511	7	Male	Trigonocephaly	Metopic suture	Coronal suture	Antigen identification and western blot analysis (untreated)
558	14	Male	Anterior plagiocephaly	Coronal suture	Sagittal suture	Western blot analysis (untreated) and mineralization analyses
627	11	Male	Trigonocephaly	Metopic suture	Coronal suture	Western blot analysis (untreated and treated with rapamycin) and mineralization analyses
350	12	Female	Anterior plagiocephaly	Coronal suture	Coronal suture	Western blot analysis (untreated) and mineralization analyses
198	12	Male	Scaphocephaly	Sagittal suture	Coronal suture	Western blot analysis (untreated and treated with rapamycin)
550	9	Female	Trigonocephaly	Metopic suture	Coronal suture	Western blot analysis (untreated and treated with rapamycin)
183	10	Female	Trigonocephaly	Metopic suture	Coronal suture	For qPCR
863	13	Male	Anterior plagiocephaly	Coronal suture	Coronal suture	For qPCR and western blot analysis (untreated and treated with baf-A1)
056	9	Male	Scaphocephaly	Sagittal suture	Coronal suture	For qPCR and western blot analysis (untreated and treated with baf-A1)
723	11	Female	Scaphocephaly	Sagittal suture	Coronal suture	Western blot analysis (untreated and treated with baf-A1)
508	7	Male	Scaphocephaly	Sagittal suture	Coronal suture	Western blot analysis (untreated and treated with wortmannin and rapamycin) and mineralization analyses
753	11	Female	Anterior plagiocephaly	Coronal suture	Coronal suture	Western blot analysis (untreated and treated with wortmannin and rapamycin) and mineralization analyses
217	11	Female	Scaphocephaly	Sagittal suture	Coronal suture	Western blot analysis (untreated and treated with wortmannin and rapamycin) and mineralization analyses

### SMCs isolation and culture

The methods for SMCs isolation and culture are described in previous reports^11^. In brief, the suture complex, consisting of the suture mesenchyme plus 3 mm of bone on either side of unfused sutures or the fused bony ridge plus 3 mm of bone on either side of fused sutures, was dissected from all specimens, and the overlying pericranium was removed. Calvarial specimens were then washed extensively in phosphate-buffered saline (PBS) supplemented with 1% penicillin/streptomycin (Sigma-Aldrich, St. Louis, MO, USA), cleaned of any soft tissue residues, minced into tiny pieces and blocks, and placed into 100-mm-diameter plates. When culturing cranial SMCs, tissue pieces and blocks were maintained in standard culture medium (Dulbecco’s modified Eagle medium) supplemented with 10% fetal calf serum, L-glutamine (1 g/L; Life Technologies, Grand Island, NY, USA), and 1% penicillin/streptomycin, and were allowed to attach to the culture flasks. Cells were incubated as a monolayer in a humidified atmosphere containing 5% CO_2_ at 37 °C. When cells reached 60–80% confluence, they were subcultivated for multiple passages (spending <15 days in primary culture) and used in experiments at confluence. The culture medium was replaced every 3 days.

### Osteogenic differentiation and mineralization analyses

To assess protein expression during osteogenic differentiation, SMCs were cultured to 60–80% confluence in standard culture medium, and then maintained for either 3 or 7 days in differentiation medium comprising Dulbecco’s modified Eagle medium supplemented with 10% fetal calf serum, L-glutamine (1 g/L), 1% penicillin/streptomycin, CaCl_2_ (calcium chloride, 1.4 mM), ascorbic acid (50 mg/mL), and ß-glycerophosphate (50 mg/mL).

To test the osteogenic potential of SMCs and the effect of rapamycin and wortmannin treatments, cells were seeded in 24-well plates at a density of 50,000 cells/well in 600 μL of medium. After reaching 70% confluence, cells were treated with new standard culture medium or with rapamycin or wortmannin for 24 h. Then, culture medium was replaced with differentiation medium. Mineralization was analyzed after culturing in differentiation medium for 14 days. Culture medium was changed every 3 days. After washing with PBS three times, cells were fixed in 95% ethanol for 30 min and then stained with 1% Alizarin Red S2 dye (9436-25 G Amresco, Cleveland, OH, USA) in 0.05 M tris-HCl (pH 8.0). Cells were then incubated at 37 °C for 30 min and washed with deionized water, and representative photographs were taken. The formation of mineralized nodules, defined as the percentage of mineralization area, was quantified using Image J densitometry. To minimize the variation between individuals, we normalized the mineralization area by dividing the mineralization area percentage of each group by the mineralization area percentage from the corresponding untreated and unfused sample to get the relative mineralization ratio. Three independent experiments were repeated for each condition.

### Antigen identification by flow cytometry

Cell surface antigen phenotyping was performed on human SMCs. Cells from prematurely fused and unfused sutures were cultured for two passages and harvested using 0.25% trypsin. After washing in PBS, cells were incubated for 30 min at room temperature in the dark using the Human multipotent mesenchymal stromal cell (MSC) Analysis Kit (BD Pharmingen, San Diego, CA, USA) according to the manufacturer’s guidelines. The kit contained the following: mouse anti-human CD90 FITC, CD44 PE, CD105 PerCP-Cy5.5, CD73 APC, κ PE and PE hMSC Negative Cocktail (CD34 PE, CD11b PE, CD19 PE, CD45 PE, and HLA-DR PE), and PE hMSC Negative Isotype Control Cocktail (mIgG1, κ FITC, mIgG1, κ PerCP-Cy5.5, mIgG1 κ APC mIgG1, κ PE, and mIgG2a). Cell status was determined using a FACS flow cytometer (BECTON DICKINSON, Newark, NJ, USA) and analyzed using FlowJo VX software.

### RNA extraction, reverse transcription, and PCR

RNA was extracted using TRIZOL Reagent (Life, CA) according to the manufacturer’s protocol, and then 1 µg purified RNA was reverse transcribed into cDNA using a PrimeScript RT Reagent Kit (Takara, Japan) according to the manufacturer’s instructions. Quantitative PCR was performed using Power SYBR Green PCR Master Mix (Applied Biosystems, UK) and the primers are listed in Table 2.

**Table 2:** PCR analysis: Genes and primer sequences.

Gene	Forward Primers	Reverse Primers
CD73	GGCTCCTCTCAATCATGCCG	CCAGAACATTTCATCCGTGTGT
CD90	TCGCTCTCCTGCTAACAGTCT	CTCGTACTGGATGGGTGAACT
CD105	AGCCCCACAAGTCTTGCAG	GCTAGTGGTATATGTCACCTCGC
ALP	CGTGGCTAAGAATGTCATCATGTT	TGGTGGAGCTGACCCTTGA
BSP	TTTCTGCTACAACACTGGGCTATG	TTGAGAAAGCACAGGCCATTC
COL1A1	CGAAGACATCCCACCAATCAC	TTGTCGCAGACGCAGATCC
GAPDH	ACAACTTTGGTATCGTGGAAGG	GCCATCACGCCACAGTTTC

### Evaluation of cell viability

A 3-(4,5-dimethylthiazol-2-yl)−2,5-diphenyltetrazolium bromide (MTT) assay kit (Sigma, Steinheim, Germany) was used to assess cell viability as follows. Cells were seeded at a density of 10,000 cells/well in 100 μL of medium in 96-well plates. Cells were then treated with rapamycin or wortmannin for 24 h, or bafilomycin A1 (BafA1) for 3 h. After treatment, MTT solution was added to the culture medium to a final concentration of 500 μg/mL, and the cells were incubated for a further 2 h at 37 °C in the dark. The supernatant was then removed, dimethyl sulfoxide was added to the wells to dissolve the formazan formed during the MTT assay, and the plates were shaken gently at 37 °C for 15 min. The absorbance of the resulting solution was measured at 570 nm using the GloMax-Multi Detection System (Promega, Madison, WI, USA). Rapamycin and wortmannin were purchased from Sigma-Aldrich (St. Louis, MO, USA), and BafA1 was purchased from Cayman Chemical Company (Ann Arbor, MI, USA).

### Western blot analysis

Cells were washed twice with cold PBS and then harvested by trypsinization with 0.25% trypsin. For total protein isolation, cells were lysed in lysis buffer (Beyotime, Haimen, China) on ice for 30 min and centrifuged at 13,400 × *g* for 30 min at 4 °C. Total protein concentration was determined using a bicinchoninic acid protein assay kit (Bio-Rad, Hercules, CA, USA) according to the manufacturer’s instructions, and then equal amounts of protein were separated by 10% sodium dodecyl sulfate-polyacrylamide gel electrophoresis and transferred to a nitrocellulose filter membrane. After blocking in tris-buffered saline (pH7.5) containing 0.1% tween 20 (TBST) and 5% nonfat milk for 1 h, membranes were incubated with primary antibodies (1:1000 dilution) overnight at 4 °C. After washing with TBST, the membrane was incubated for 30 min at room temperature with an IRDye 800-conjugated goat anti-rabbit IgG secondary antibody (Rockland Immunochemicals, Limerick, PA, USA; 1:2000 dilution). Protein bands were visualized using a Licor Odyssey Infrared Imaging System (LI-COR Biosciences, Lincoln, NE, USA). Antibodies against β-actin were purchased from Cell Signaling Technology (Danvers, MA, USA). Anti-LC3-II antibodies were purchased from Novus Biologicals (Littleton, CO, USA).

### Statistical analyses

Results are presented as the mean ± standard deviation (SD). The significance of differences among groups was determined by one-way ANOVA followed by Tukey’s post-hoc test. Statistical significance was defined as described in the figure legends.

## Results

### Mesenchymal cell populations isolated from fused and unfused cranial sutures are identical

**Figure 1:**
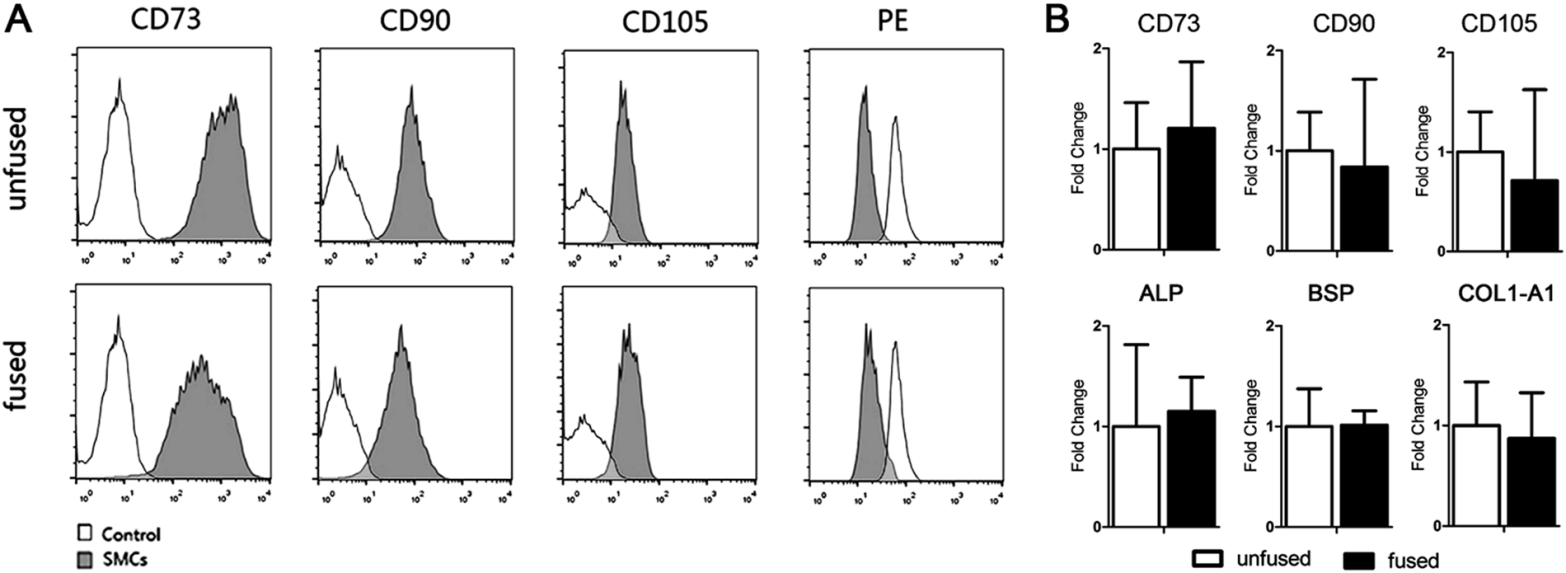
Characterization of the fused and unfused cranial SMCs. (**A**) Cell surface antigen phenotyping of human calvarial SMCs. Flow cytometric analysis showing the surface expression of CD90, CD105, and CD73, indicative of a mesenchymal immunophenotype, on both fused and unfused SMCs. PE: PE-labeled negative multipotent mesenchymal stromal cell (MSC) cocktail; control: negative control group. (**B**) Expression of the mesenchymal markers CD90, CD105, and CD73, as well as expression of the osteogenic markers ALP and BSP, and the chondrogenic marker COL1-A1 in fused and unfused SMCs, as determined by qPCR. Data are expressed as the mean ± SD of three independent experiments.

Abnormal osteogenic activity in cranial SMCs is generally considered a common cause of craniosynostosis. Hence, we isolated mesenchymal cells from the fused and unfused sutures of patients with nonsyndromic monosutural craniosynostosis to investigate disease pathogenesis. Flow cytometry was first used to characterize the identity of isolated cell populations. Results showed that greater than 90% of SMCs from both fused and unfused sutures expressed typical human MSC markers including CD90, CD105, and CD73 (Fig. 1A). Expression levels of these markers were similar between fused and unfused SMCs (Fig. 1B). To further characterize these cells, we examined the expression of the osteogenic markers ALP, BSP, and COL1-A1 by qPCR. Indeed, these markers were expressed in isolated SMCs and no significant differences in expression levels were observed between the fused and unfused cranial SMCs, indicating that they have osteogenic capability and exist in a similar osteogenic differentiation state (Fig. 1B). However, the isolated fused and unfused SMCs gradually lost growing capability within 2 months.

### Autophagic activity is enhanced in SMCs from the fused suture

**Figure 2:**
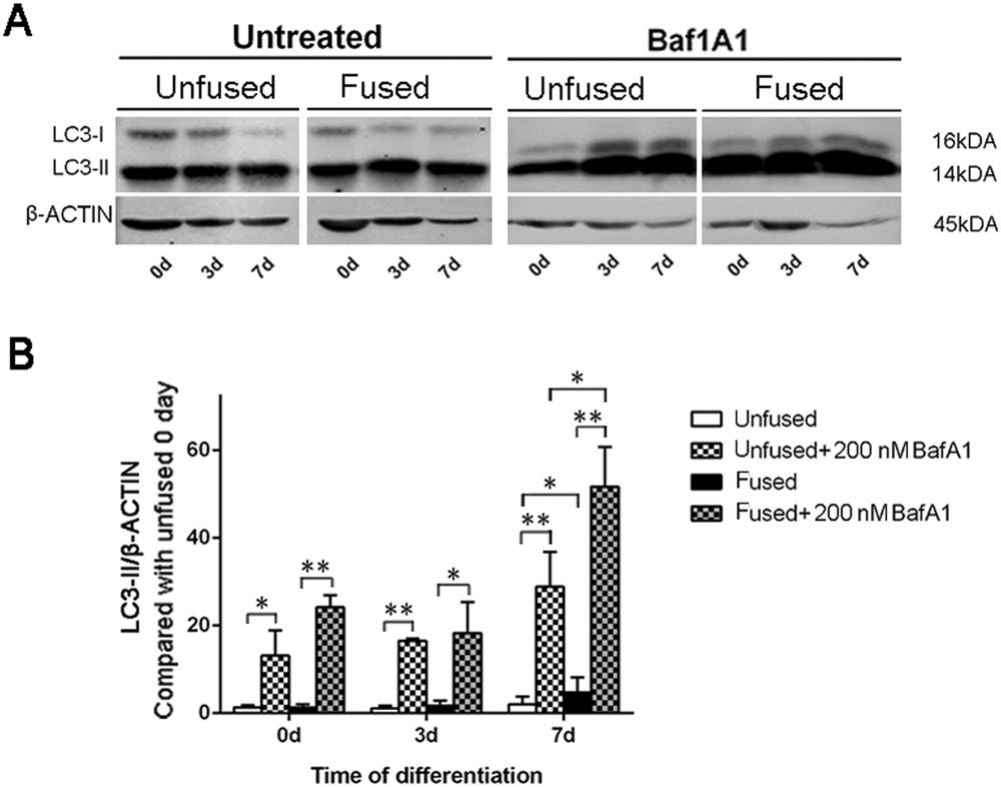
Expression of the autophagosomal marker LC3-II in fused and unfused cranial SMCs. (**A**) Representative western blot of fused and unfused SMCs after 0, 3, and 7 days in osteogenic culture medium. To evaluate autophagic flux, BafA1 was used to block autophagosome and lysosome fusion. Full-length blots are presented in Supplementary Fig. 1. (**B**) Quantification of protein expression by densitometry. Full-length blots are presented in Supplementary Figure 2. Data are expressed as the mean ± SD of three independent experiments. *P < 0.05; **P < 0.01.

Autophagy has been shown to be essential for bone mineralization^28^. Therefore, we hypothesized that hyperactive autophagy might play a role in the premature calvarial ossification that leads to craniosynostosis. To this end, we examined the level of LC3-II protein, a common marker for evaluating autophagic activity, during the osteogenic differentiation of SMCs. Consistent with a previous report using the UMR-106 osteoblastic cell line^28^, LC3-II protein levels were not changed in SMCs at differentiation day 3, but were significantly increased at differentiation day 7 (Fig. 2A,B). As expected, we noted that LC3-II levels in SMCs from the fused suture were higher than those of SMCs from the unfused suture at differentiation day 7 (Fig. 2A,B). This suggests that fused SMCs have the propensity for enhanced autophagy during osteogenesis.

To investigate whether increased LC3-II in the fused SMCs was due to abnormal autophagosome production or autophagosomal consumption, we treated the cells with the specific lysosomal proton pump inhibitor BafA1, which can block autophagosome/lysosome fusion. Indeed, inhibition of autophagosome maturation led to a marked increase in LC3-II levels in both fused and unfused SMCs, irrespective of osteogenic differentiation induction. However, this increase was particularly striking in the fused SMCs at differentiation day 7, which was significantly higher than that in unfused SMCs at the same stage of osteogenic differentiation (Fig. 2A,B). These results indicate that SMCs in the fused suture exhibit enhanced autophagosome production during osteogenic differentiation.

### Induction or inhibition of autophagy can enhance or repress the osteogenic differentiation of SMCs

**Figure 3:**
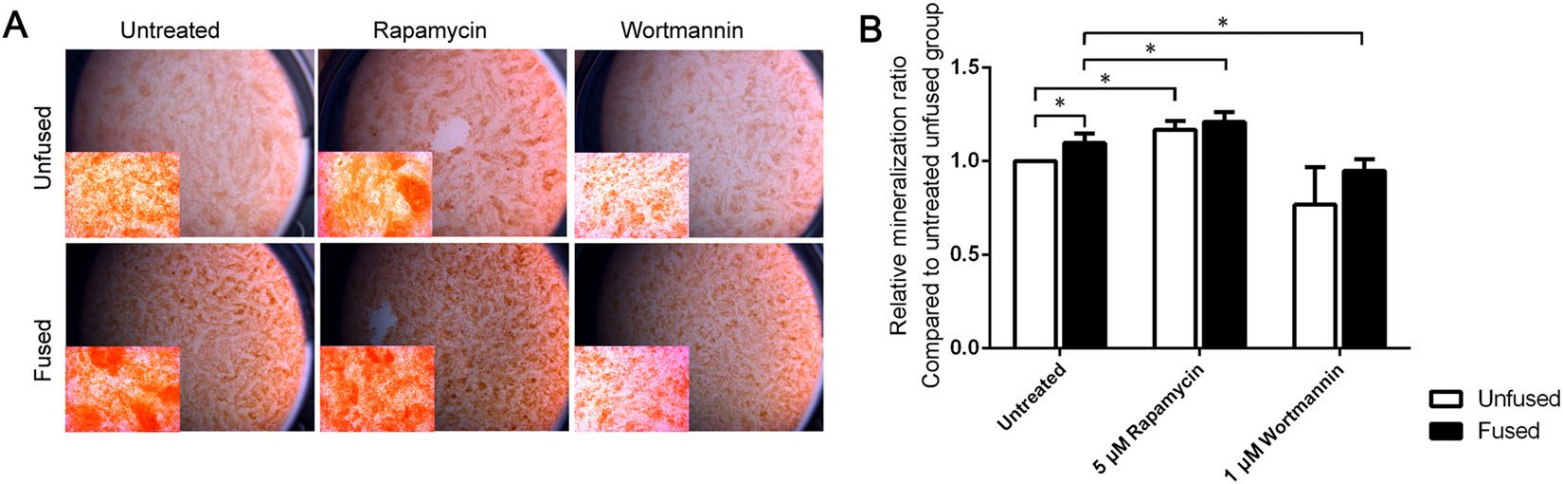
Effect of the modulation of autophagy on cranial SMC mineralization. (**A**) Representative Alizarin Red S2 staining of mineralization nodules. Samples were cultured in osteogenic medium for 14 days before staining. Insets in each panel are magnified views. (**B**) Quantification of mineralization. To normalize individual differences, the relative mineralization ratio was calculated for comparison. Details are described in the Materials and Methods section. Data are expressed as the mean ± SD of three independent experiments. *P < 0.05.

Having determined that the fused SMCs exhibit enhanced autophagy during osteogenic differentiation, we wondered whether this abnormality is associated with enhanced ossification. After 2 weeks of differentiation, calvarial SMCs were stained with Alizarin Red S2 to reveal bone mineralization. Indeed, the fused SMCs formed more bone nodules than the unfused SMCs (Fig. 3A,B). Moreover, nodules from the fused SMCs stained darker (Fig. 3A), suggesting enhanced mineralization. These data indicate the fused SMCs have augmented osteogenic capability.

To determine if disturbed autophagy can affect osteogenesis in SMCs, we treated the cells with the autophagy inducer rapamycin (5 µM) or the autophagy inhibitor wortmannin (1 µM) for 24 h before transferring them to osteogenic differentiation medium. After 14 days in osteogenic differentiation medium, SMCs was stained with Alizarin Red S2. Indeed, rapamycin treatment significantly enhanced bone nodule formation in both the fused and unfused groups (Fig. 3A,B), whereas wortmannin treatment significantly decreased osteogenic activity in both groups (Fig. 3A,B). These results indicate that autophagy is essential for the osteogenesis of human SMCs. However, we did not note a significant difference in bone nodule formation between the fused and unfused groups with either rapamycin or wortmannin treatment (Fig. 3A,B).

**Figure 4:**
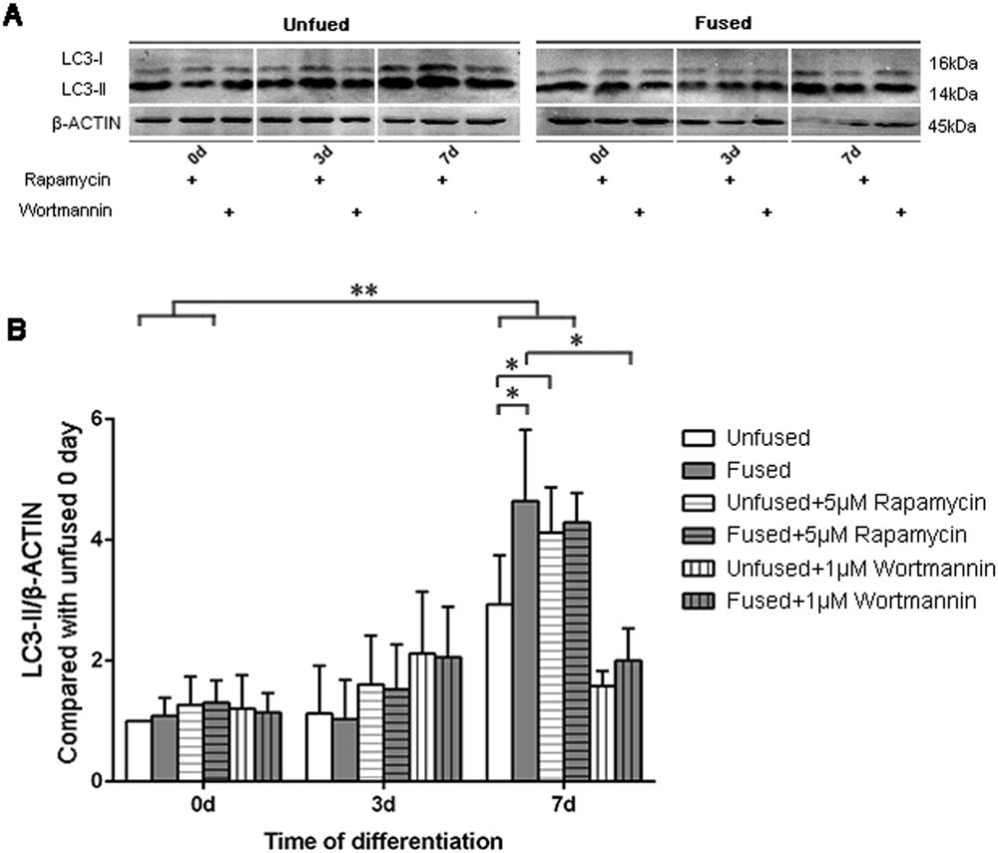
Effect of rapamycin or wortmannin on autophagic induction during cranial SMC differentiation. (**A**) Representative western blot of fused and unfused SMCs treated with or without rapamycin or wortmannin at different differentiation time points. (**B**) Quantification of LC3 and β-actin expression level by densitometry. Full-length blots are presented in the Supplementary Fig. 3. Data are expressed as the mean ± SD of three independent experiments. *P < 0.05; **P < 0.01.

To verify the effect of rapamycin or wortmannin on autophagic activity, we examined LC3-II protein expression in treated SMCs at differentiation day 7. As expected, LC3-II was increased in response to rapamycin in both the fused and unfused groups, whereas wortmannin reduced the levels of this protein in both groups (Fig. 4A,B). Consistent with the effect of rapamycin and wortmannin on ossification outcome, we did not identify a significant difference in LC3-II protein levels between the fused and unfused groups after treatment with rapamycin or wortmannin.

## Discussion

NSC is a common inborn heteroplasia. Recently, local tissue-specific differences in the activity of cellular networks have been associated with intramembranous calvarial ossification^11^. FGF signaling, an important regulatory factor related to craniosynostosis, regulates bone growth through autophagy^24^. Thus, we proposed a new idea whether there was a relationship between autophagy and local premature suture ossification. Although mandible-derived bone MSCs exhibit stronger autophagy and anti-aging capacities than tibia-derived bone MSCs^22^, the role of local differences in autophagy in the pathogenesis of premature suture ossification is poorly understood. Here, we confirm that autophagic activity is involved in the development and maintenance of cranial sutures, and reveal that autophagy plays a role in premature suture ossification. Our findings provide evidence of a potentially novel mechanism for NSC development.

Autophagy is a major catabolic process required for the maintenance of tissue homeostasis, particularly under conditions of stress^22^. It has been shown to be involved in various aspects of bone growth and health^29^. Here we show that autophagy is also essential for osteogenesis in human SMCs. As previously reported for the UMR-106 cell line and rodent primary osteoblasts, we observed that LC3-II levels are not changed during the early differentiation stage. A significant increase in LC3-II protein levels was not observed until differentiation day 7, which represents a time point after the initiation of mineralization^28^. This suggests that the induction of autophagy is associated with SMC ossification. Modulation of autophagy using rapamycin or wortmannin indeed enhanced or repressed SMC ossification, respectively. Our findings indicate that the induction of autophagy is also required for mineralization during human SMC differentiation. In addition to fusion of the autophagosome with the lysosome to degrade its content, exocytosis of the autophagic vacuole was previously shown to be an unconventional protein secretion pathway^21^. Studies have shown that mineralization can be initiated with cells^30^. Therefore, it has been proposed that autophagic vacuoles serve as a pathway to secret apatite crystals from osteoblasts^28^. Our work suggests that this mineralization mechanism might also be conserved in human cells.

During osteogenic differentiation, autophagic induction in the fused SMCs is activated to a level higher than that in the unfused SMCs, and consequently, more mineralized nodules were observed in the fused group after 14 days of culture in mineralizing conditions. Our findings indicate that overactive autophagy is a novel pathological mechanism resulting in premature ossification in NSC patients. It is notable that the fused and unfused suture tissues in this study were obtained from the same NSC patient. Hence either somatic mutations or exogenous factor-induced epigenetic changes might be the underlying cause. Our work suggests that mutations or epigenetic changes in autophagy-related genes should be explored to understand NSC etiology.

To verify the role of autophagy in regulating the osteogenic differentiation of SMCs, we used rapamycin and wortmannin to modulate autophagy. Although we noted that activation or inhibition of autophagy could significantly promote or repress mineralization in both types of SMCs, we did not observe a difference between the fused and unfused SMCs after rapamycin or wortmannin treatment. One possibility is that these treatments might function downstream of primary pathological defects, which cancels out the upstream difference. Alternatively, the effect of rapamycin or wortmannin treatment may be so dramatic that it overrides the difference caused by primary pathological defects.

Overall, our data revealed the existence of autophagy during the osteogenic differentiation of human calvarial SMCs and that overactive autophagy in pathological calvarial SMCs is associated with premature calvarial ossification in NSC. Thus, inhibition of autophagy could be a promising approach to treat NSC.

## Electronic supplementary material

Supplementary Information

Supplementary Figure 1

Supplementary Figure 2

Supplementary Figure 3
