# Supplementary material for: Overactive autophagy is a pathological mechanism underlying premature suture ossification in nonsyndromic craniosynostosis
Source: Sci Rep. 2018 Apr 25;8:6525. doi: 10.1038/s41598-018-24885-z (PMC5916928; doi:10.1038/s41598-018-24885-z)
Supplement: Supplementary file 1 — Supplementary Information [file 41598_2018_24885_MOESM1_ESM.docx]

**Supplementary Figure 1. Full-length blots are presented from Figure 2.** Representative Western blot of the fused and unfused cranial suture mesenchymal stromal cells (SMCs) after 0, 3, 7 and 10 days in osteogenic culture medium.

**Supplementary Figure 2. Full-length blots are presented from Figure 2.**

**Supplementary Figure 3. Full-length blots are presented from Figure 4.**
