# Supplementary figures and images for: Overactive autophagy is a pathological mechanism underlying premature suture ossification in nonsyndromic craniosynostosis
Source: Sci Rep. 2018 Apr 25;8:6525. doi: 10.1038/s41598-018-24885-z (PMC5916928; doi:10.1038/s41598-018-24885-z)

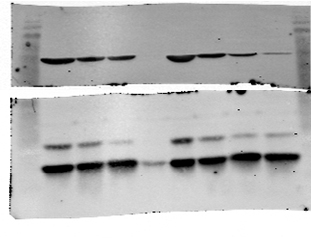

Supplement: Supplementary file 2 — Supplementary Figure 1 [file 41598_2018_24885_MOESM2_ESM.tif]

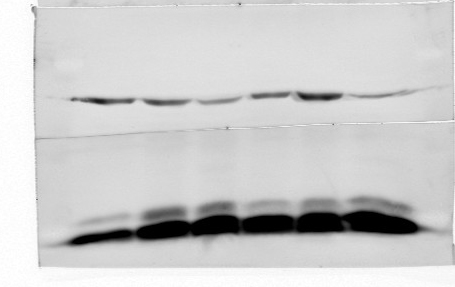

Supplement: Supplementary file 3 — Supplementary Figure 2 [file 41598_2018_24885_MOESM3_ESM.tif]

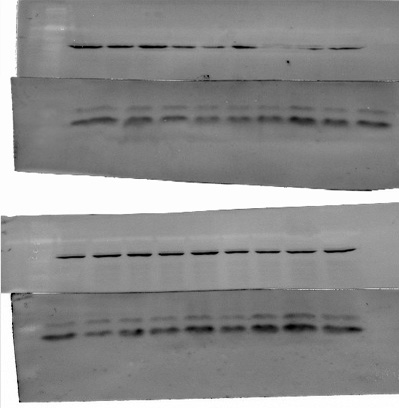

Supplement: Supplementary file 4 — Supplementary Figure 3 [file 41598_2018_24885_MOESM4_ESM.tif]
